# Long-Term Oncological Outcomes of Laparoscopic Versus Open Radical Surgery in Early-Stage Cervical Cancer: A Propensity Score–Matched Analysis

**DOI:** 10.3390/cancers17243960

**Published:** 2025-12-11

**Authors:** Rattiya Phianpiset, Chayanid Detwongya, Sunisa Phookiaw, Manatsawee Manopunya, Chailert Phongnarisorn, Kittipat Charoenkwan

**Affiliations:** Division of Gynecologic Oncology, Department of Obstetrics and Gynecology, Faculty of Medicine, Chiang Mai University, Chiang Mai 50200, Thailand; rattiya.phi@cmu.ac.th (R.P.); chayanid.png@gmail.com (C.D.); sunisa.phookiaw@gmail.com (S.P.); manatsawee.m@cmu.ac.th (M.M.); chailert.p@cmu.ac.th (C.P.)

**Keywords:** cervical cancer, laparoscopy, radical hysterectomy, propensity score matching, overall survival, progression-free survival

## Abstract

**Objective**: To assess the oncological outcomes of laparoscopic versus open radical hysterectomy (RH) in patients with early-stage cervical cancer using propensity score–matched analysis. **Methods**: We conducted a retrospective cohort study of 1244 patients who underwent RH with pelvic lymphadenectomy at Chiang Mai University Hospital between 2003 and 2019. Of these, 82 patients received a laparoscopic approach (LAP) and 1162 underwent open radical hysterectomy. Propensity-score matching was performed in a 1:4 ratio using a caliper of 0.2 standard deviations to achieve balance between groups. Overall survival (OS) and progression-free survival (PFS) were analyzed with Kaplan–Meier curves and the log-rank test. Subgroup analysis was conducted based on tumor size (≤ 2 cm vs. > 2 cm). In addition, multivariable Cox proportional hazards models incorporating all relevant clinical and pathological variables were applied to the overall cohort to assess independent predictors of OS and PFS. **Results**: After matching, 72 LAP RH cases were compared with 279 open RH cases, showing well-balanced baseline features. At 5 years, OS was nearly the same between the LAP and the open groups (95.8% vs. 95.5%; *p* = 0.95), and PFS was also similar (92.3% vs. 93.8%; *p* = 0.85). Subgroup analyses demonstrated that LAP RH did not result in a survival disadvantage for tumors ≤ 2 cm or > 2 cm. In multivariable Cox analysis, surgical approach was not an independent predictor of (HR 0.83, 95% CI 0.40–1.71, *p* = 0.61) or PFS (HR 1.12, 95% CI 0.44–2.84, *p* = 0.82). **Conclusions**: In our single-center cohort analyzed using propensity score matching, LAP RH showed long-term oncological outcomes comparable to those of open RH. These results support LAP RH as a safe surgical option for selected patients with early-stage cervical cancer within our setting, where procedures were performed by experienced surgeons following standardized techniques. Further evaluation in diverse clinical contexts is still needed.

## Simple Summary

Radical hysterectomy is a standard treatment for early-stage cervical cancer and can be performed through open or minimally invasive surgery. After reports suggesting that minimally invasive approaches may worsen survival, their safety has been questioned worldwide. This study compared long-term survival outcomes between laparoscopic and open radical hysterectomy in women with early-stage cervical cancer treated at a high-volume center. Using propensity-score matching and multivariable analyses, we found no significant differences in five-year overall or progression-free survival between the two surgical methods, even after adjusting for tumor size. These results suggest that, when performed by experienced surgeons using refined techniques and careful patient selection, laparoscopic radical hysterectomy can achieve oncologic outcomes equivalent to open surgery. Our findings may help guide future surgical decision-making and support ongoing efforts to optimize minimally invasive approaches for cervical cancer treatment.

## 1. Introduction

Radical hysterectomy (RH) with pelvic lymphadenectomy has long been the standard of care for women with early-stage cervical cancer [1]. The procedure is traditionally executed via an open abdominal approach. This conventional technique, while oncologically effective, is often accompanied by considerable morbidity, including substantial blood loss, increased risk of postoperative complications, and extended recovery periods [2]. In recent years, the advent of minimally invasive surgical (MIS) techniques has gained interest in the field of gynecologic oncology due to their potential to mitigate these adverse effects while preserving oncological outcomes. Observational studies and meta-analyses comparing MIS to the traditional open approach for RH in early-stage cervical cancer initially suggested comparable oncological outcomes with added benefits of reduced perioperative morbidity, shorter hospitalization, and faster postoperative recovery, favoring MIS [3,4]. However, the Laparoscopic Approach to Cervical Cancer (LACC) study, a multicenter phase III randomized controlled trial comparing the MIS vs. the open approach for RH in early-stage cervical cancer, has challenged these perceived benefits by demonstrating that MIS RH may be linked to inferior survival outcomes when compared with the open approach. [5]. Specifically, the 4.5-year disease-free survival was 86.0% in the MIS group versus 96.5% in the open group, with a nearly four-fold increased risk of recurrence or death associated with MIS. These results led many institutions and guidelines to recommend open surgery as the preferred approach [6].

Nonetheless, controversy persists. Several large retrospective and population-based studies have reported conflicting outcomes. For instance, some recent studies from Asia showed no survival disadvantage of MIS when controlling for tumor characteristics [7,8]. Some reports also suggest that detrimental outcomes associated with MIS may be confined to tumors > 2 cm or related to specific technical factors, such as the use of uterine manipulators or failure to close the vaginal cuff before colpotomy [9]. Others argue that the LACC trial’s findings may not be generalizable due to limited surgeon experience, relatively small sample size, and the rapid evolution of MIS technology [10].

This ongoing debate underscores the need for further analysis. Notably, comparisons between MIS and open surgery in real-world cohorts are often complicated by differences in patient and tumor characteristics, surgeon expertise, and postoperative treatment. Such confounding factors can mask the true oncological effect of the surgical approach. To overcome this issue, propensity score-based methods offer a robust statistical tool to balance baseline differences between groups and simulate randomized comparisons. Propensity-score matching is a practical and widely accepted method for reducing bias in observational studies. By estimating the likelihood that a patient would undergo MIS versus open surgery based on observed clinical and tumor variables, propensity-score matching creates comparable groups where these baseline differences are minimized. This method decreases selection bias from non-random treatment assignments and ensures that subsequent comparisons reflect outcomes more directly related to the surgical approach rather than underlying prognostic differences. Furthermore, when applied carefully with suitable covariate selection, matching algorithms, and balance checks, the techniques can approximate the counterfactual framework of randomized controlled trials. In gynecologic oncology, where randomized evidence is often limited or ethically difficult to obtain, propensity-score matching improves causal inference and increases the reliability of real-world comparative effectiveness analyses [11].

Therefore, the present study aimed to evaluate the long-term oncological outcomes of laparoscopic (LAP) versus open radical surgery for early-stage cervical cancer in a high-volume center, where procedures are performed by experienced gynecologic oncologists using standardized techniques. We employed propensity score–matched analyses to control for confounding factors. The results may contribute to the global evidence base and inform surgical decision-making for women with early cervical cancer, particularly in the Asian population, where disease burden remains high.

## 2. Materials and Methods

### 2.1. Study Design and Patient Population

We conducted a retrospective cohort study of patients with early-stage cervical cancer who underwent radical surgery at Chiang Mai University Hospital between January 2003 and December 2019. Eligible patients were those who (1) had histologically confirmed squamous cell carcinoma, adenocarcinoma, or adenosquamous carcinoma of the cervix; (2) had FIGO 2009 stage IA1 with lymphovascular space invasion (LVSI), IA2, IB1, IB2, or IIA disease, assessed preoperatively; (3) underwent Querleu-Morrow Type B or C RH with pelvic lymphadenectomy [12,13]; (4) had follow-up available for 3 months or longer, or until an oncological event (recurrence or death). We excluded patients who (1) received neoadjuvant chemotherapy or radiotherapy before surgery; (2) underwent non-radical or palliative surgery; (3) had incomplete or missing critical data, including survival outcomes; (4) had cervical cancer during pregnancy.

The Research Ethics Committee of the Faculty of Medicine, Chiang Mai University approved this study (approval number: 370/2568, 12 September 2025).

### 2.2. Exposure and Outcomes

The exposure was the surgical approach, defined as LAP versus open radical surgery. At our institution, laparoscopic procedures were performed using a standard 4–5-port approach by two senior gynecologic oncologists (CP and KC), each of whom had experience with more than 200 radical surgeries. Open radical hysterectomy was performed by 12 board-certified gynecologic oncologists, including CP and KC.

Postoperative adjuvant therapy was delivered according to institutional protocols that aligned with standard risk-based criteria. Patients exhibiting high-risk pathological features including pelvic lymph node involvement, parametrial invasion, or positive vaginal margins were treated with concurrent chemoradiation (CCRT), consisting of pelvic external beam radiotherapy at 50.4 Gy in 28 fractions and weekly cisplatin at 40 mg/m^2^ for 4–6 cycles [14]. Those meeting intermediate-risk criteria determined by the Sedlis system, which combined three factors—LVSI, depth of stromal invasion, and tumor size—received adjuvant radiotherapy [15]. After completing treatment, follow-up visits were scheduled every three months in the first year, every four months in the second year, every six months from years three to five, and annually thereafter.

The primary oncological outcomes were overall survival, defined as time from surgery to death from any cause, and progression-free survival, defined as time from surgery to first documented recurrence, diagnosed according to the Response Evaluation Criteria in Solid Tumors (RECIST) [16]. Patients without recurrence or death were censored at the last follow-up. Survival outcomes were compared between the LAP and the open groups, with statistical models adjusted for potential confounders, including demographic, clinicopathological, and treatment-related covariates.

### 2.3. Covariates

Candidate covariates were selected based on clinical relevance and existing research. Covariates examined for baseline balance and potential inclusion included demographics (age, parity, human immunodeficiency virus [HIV] infection), tumor characteristics (tumor size, histology, grade, depth of cervical stromal invasion, LVSI), cancer extent (vaginal metastasis, uterine corpus invasion, adnexal metastasis, parametrial invasion, and pelvic/para-aortic node metastasis), and treatment (prior conization, RH type [B/C], number of dissected lymph nodes, surgical margins, and adjuvant therapy).

We assessed multicollinearity among candidate covariates using Pearson correlation coefficient for continuous variables and Cramér’s V for categorical variables. When two variables were highly correlated, only one was retained in the final covariate set. Standardized mean differences (SMDs) were used to quantify baseline between-group imbalance in covariates, with |SMD| > 0.10 indicating significant imbalance. Subsequently, the imbalanced covariates were included in the propensity-score model to control for confounding.

### 2.4. Handling of Missing Data

Patterns and proportions of missing data were examined (Table S1). Variables with over 15% missing values, including tumor grade, LVSI, and depth of invasion, were excluded from the propensity score model due to significant collinearity and to preserve sample size and reduce variance. For the remaining variables, each with less than 5% missing values, we used complete-case analysis to estimate propensity scores.

### 2.5. Propensity-Score Estimation and Matching

We estimated each patient’s probability of undergoing LAP using a logistic regression model that incorporated the remaining imbalanced covariates, after accounting for collinearity and missingness. These included pathological tumor size, histology, uterine corpus involvement, adnexal metastasis, parametrial invasion, pelvic node metastasis, prior conization, RH class, parametrial margin status, and postoperative adjuvant therapy. The propensity scores were then used for matching by employing the nearest-neighbor method. Matching was performed using a caliper of 0.2 standard deviation (SD) of the logit propensity scores, allowing up to 1:4 nearest-neighbor matches. Only complete cases for the matching variables were included. Post-match balance was evaluated with SMD (target |SMD| < 0.10) and visualized with Love plots.

### 2.6. Survival Analysis

We generated Kaplan–Meier curves to estimate OS and PFS comparing LAP versus open in the whole cohort and in the propensity-score-matched cohort. Survival outcomes differences between the LAP and the open groups were evaluated with log-rank tests, and survival probabilities at 60 months were summarized.

### 2.7. Subgroup Analysis

Subgroup analysis was performed to evaluate the relationship between surgical approaches and OS and PFS, stratified by tumor size (≤2 cm vs. >2 cm). The procedures for covariate selection, propensity score estimation/matching, and survival analysis, as outlined in Section 2.3, Section 2.4, Section 2.5 and Section 2.6, were carried out separately for patients with tumors smaller than 2 cm and those with tumors larger than 2 cm.

### 2.8. Additional Analyses

Cox proportional hazards models were employed to estimate hazard ratios. In the overall cohort, multivariable models accounting for all relevant covariates were developed for both overall survival and progression-free survival. An exploratory analysis within the LAP subgroup also assessed the effect of the no-contamination technique, defined as vaginal cuff closure without using a uterine manipulator, on oncological outcomes. These models included adjustments for age, tumor size, histology, uterine corpus invasion, parametrial involvement, pelvic nodal status, RH class, and vaginal margin status.

All analyses were performed in R version 4.5 (R Foundation for Statistical Computing, Vienna, Austria, 2025). Statistical significance was set at a two-sided *p*-value of <0.05.

## 3. Results

### 3.1. Patient Population and Baseline Characteristics

**Figure 1:**
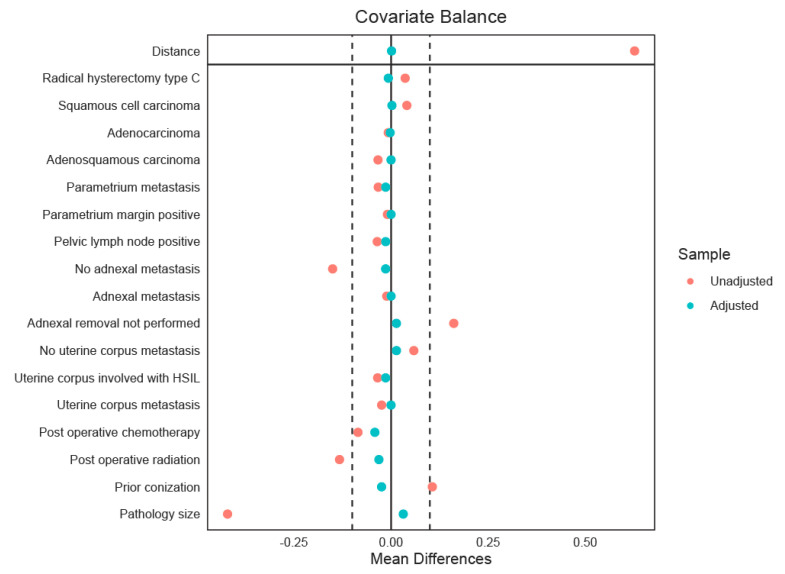
The Love plot displays the standardized mean differences (SMDs) of covariates between treatment groups before (red) and after (green) propensity-score matching. The solid vertical line at zero indicates perfect covariate balance, meaning no difference in means between groups. The dashed lines at ±0.1 mark the usual cutoff for acceptable imbalance. Covariates with SMDs within these boundaries are deemed well-balanced, showing that matching has successfully reduced baseline differences.

A total of 1244 patients met the eligibility criteria, of whom 82 (6.6%) underwent LAP and 1162 (93.4%) underwent open radical surgery. Before adjustment, patients in the LAP group had significantly smaller tumors compared with those in the open group (median 1.0 vs. 2.0 cm, *p* < 0.001). Previous conization was also more frequent among LAP patients (51.2% vs. 38.6%, *p* = 0.031). Pathological features also showed notable differences. LVSI was less frequent in the LAP group (43.9% vs. 58.1%, *p* = 0.033), and adnexal metastasis was absent in the LAP group compared with 1.1% in the open group (*p* = 0.007). In addition, patients undergoing LAP were less likely to receive postoperative chemotherapy (19.8% vs. 32.9%, *p* = 0.014) and were less likely to undergo adjuvant radiation (27.6% vs. 41.4%, *p* = 0.023). After propensity-score matching, 72 patients in the LAP group were matched to 279 patients in the open group (1:4 ratio), yielding well-balanced baseline characteristics between the groups. Standardized mean differences for all variables were below 0.10. (Table 1 and Figure 1) Of note, for the propensity-score matched cohort, operative outcomes differed, with LAP associated with a lower number of pelvic lymph nodes resected (median 20.0 vs. 26.0 nodes, *p* < 0.001), longer operative time (median 411.5 vs. 219.0 min, *p* < 0.001), but less operative blood loss (median 300 vs. 400 mL, *p* < 0.001). The overall rate of perioperative complications did not differ significantly between the two groups (12.5% in LAP vs. 10.0% in open, *p* = 0.695).

### 3.2. Survival Outcomes

Table 2 compares oncological outcomes between the two groups. The median follow-up duration was 86.0 months in the LAP RH group and 78.2 months in the open RH group.

In the overall unmatched cohort, OS did not differ significantly between patients undergoing LAP and those treated with open radical surgery. The Kaplan–Meier estimates showed nearly overlapping survival curves, with a five-year OS of 92.8% (95% confidence interval [CI] 91.2–94.3%) in the open group compared with 91.4% (95% CI 85.6–97.7%) in the LAP group (log-rank *p* = 0.91). PFS was also comparable between the two surgical approaches. The five-year PFS was 90.6% (95% CI 88.7–92.5%) in the open group and 90.5% (95% CI 84.1–97.5%) in the LAP group, with no statistically significant difference (log-rank *p* = 0.96) (Figure S1).

**Figure 2:**
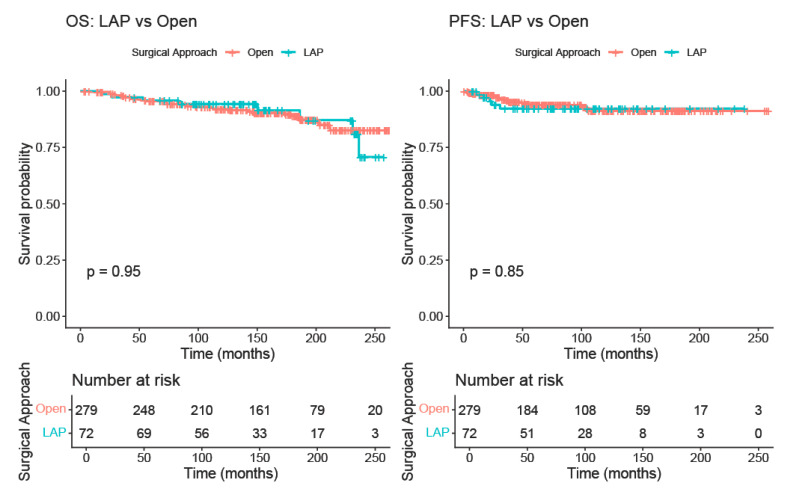
Kaplan–Meier curve comparing overall survival and progression-free survival between the LAP and the open RH groups (after matching).

After propensity-score matching, OS remained comparable between surgical approaches. Five-year OS in the open group was 95.5% (95% CI 93.0–98.0%) while in the LAP group, it was 95.8% (95% CI 91.3–100.0%). The Kaplan–Meier curves overlapped substantially, and no statistically significant difference was observed (log-rank *p* = 0.95). PFS was also similar between groups, with a five-year PFS of 93.8% (95% CI 90.7–97.1%) in the open group and 92.3% (95% CI 86.0–99.0%) in the LAP group. Again, the survival curves did not differ significantly (log-rank *p* = 0.85) (Figure 2).

In the subgroup analysis of the matched cohort stratified by pathological tumor size, both OS and PFS showed no significant differences between LAP and open RH in patients with tumors ≤ 2 cm or >2 cm. Baseline characteristics of the cohort and survival outcomes for tumors ≤ 2 cm and >2 cm are shown in Tables S2–S5, respectively.

**Figure 3:**
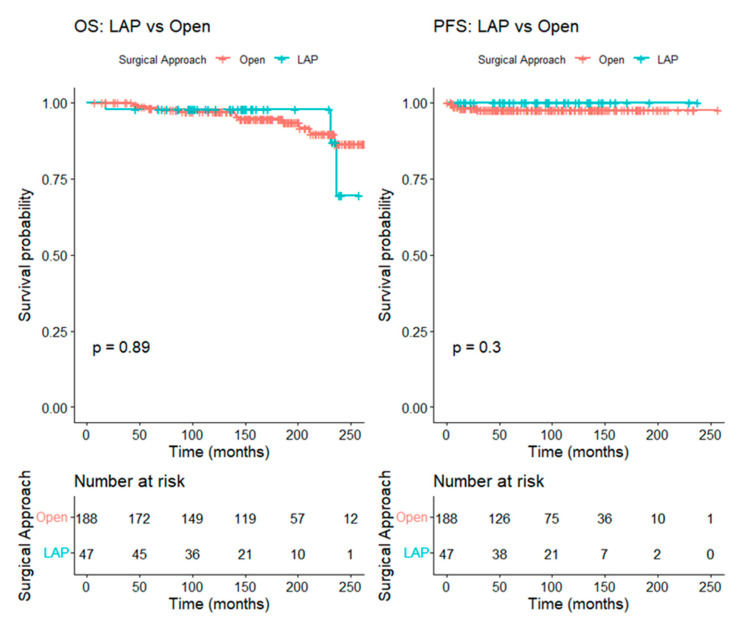
Kaplan–Meier curve comparing overall survival and progression-free survival between the LAP and the open RH groups in patients with tumors ≤ 2 cm (after matching).

For patients with tumors ≤ 2 cm, 47 patients in the LAP group were matched to 188 patients in the open group at a 1:4 ratio, resulting in well-balanced baseline characteristics between the groups. The five-year OS was 98.3% (95% CI 96.4–100.0%) in the open group and 97.9% (95% CI 93.8–100.0%) in the LAP group (log-rank *p* = 0.89), while the five-year PFS was 97.7% (95% CI 95.4–100.0%) in the open group and 100.0% (95% CI 100.0–100.0%) in the LAP group (log-rank *p* = 0.30) (Figure 3).

**Figure 4:**
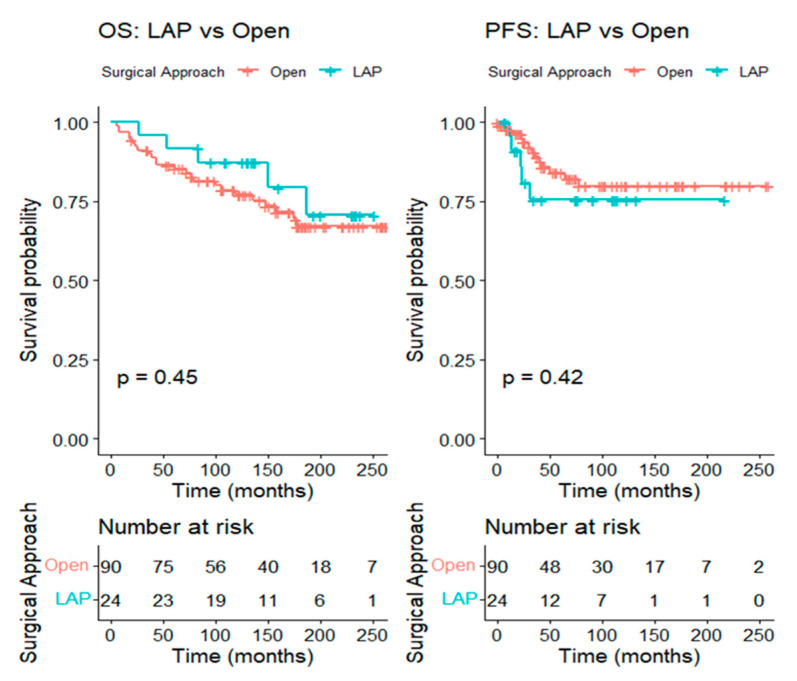
Kaplan–Meier curve comparing overall survival and progression-free survival between the LAP and the open RH groups in patients with tumors > 2 cm (after matching).

For tumors larger than 2 cm, 24 patients in the LAP group were matched to 90 patients in the open group at a 1:4 ratio, leading to well-balanced baseline characteristics between the two groups. The five-year OS was 86.5% (95% CI 79.6–93.9%) in the open group and 91.7% (95% CI 81.3–100.0%) in the LAP group (log-rank *p* = 0.45), while the five-year PFS was 84.1% (95% CI 75.8–93.3%) in the open group and 75.4% (95% CI 58.7–96.8%) in the LAP group (log-rank *p* = 0.42) (Figure 4).

### 3.3. Multivariable Cox Regression

In multivariable Cox regression adjusting for demographic, clinicopathologic, and treatment-related covariates, the surgical approach was not associated with death (hazard ratio [HR] 0.83, 95% CI 0.40–1.71, *p* = 0.61) or recurrence (HR 1.12, 95% CI 0.44–2.84, *p* = 0.82).

For OS, independent adverse prognostic factors included increasing age (HR 1.03, 95% CI 1.00–1.05, *p* = 0.02), adenosquamous histology (HR 1.77, 95% CI 1.09–2.88, *p* = 0.02), pathological tumor size (HR 1.34, 95% CI 1.16–1.55, *p* < 0.001), pelvic lymph node metastasis (HR 1.60, 95% CI 1.02–2.51, *p* = 0.04), and vaginal metastasis (HR 1.53, 95% CI 1.01–2.31, *p* = 0.05). For PFS, significant predictors were parity (HR 0.77 [protective], 95% CI 0.62–0.95, *p* = 0.02), adenosquamous histology (HR 2.08, 95% CI 1.14–3.82, *p* = 0.02), and pathological tumor size (HR 1.69, 95% CI 1.42–2.03, *p* < 0.001) (Table 3).

Exploratory analysis of 82 patients who underwent LAP RH showed that 43 patients (52.4%) underwent surgery with preventive measures to minimize tumor contamination, including avoiding the use of a uterine manipulator and performing vaginal cuff closure. The remaining 39 patients (47.6%) did not receive these preventive techniques. Four patients were excluded from the multivariable models due to missing data, leaving 78 evaluable cases.

Among 78 patients treated with MIS, 10 deaths and 6 recurrences were observed. In a multivariable Cox regression for OS, pathological tumor size remained a strong prognostic factor, with each 1 cm increase associated with a more than two-fold higher risk of death (HR 2.68, 95% CI 1.35–5.31, *p* < 0.01). Of note, the preventive measures for tumor contamination were not significantly associated with improved survival (HR 0.90, 95% CI 0.13–6.27, *p* = 0.92).

For PFS, type C RH was associated with lower recurrence risk (HR 8.21 × 10^−5^; 95% CI 2.21 × 10^−8^–0.30, *p* = 0.03). No other covariates demonstrated statistically meaningful associations with PFS (Table S6). To assess whether improvements in surgical performance associated with the learning curve influenced oncologic outcomes, we evaluated the surgery period (2003–2008 vs. 2009–2019) as a covariate. Surgery period was not significantly associated with OS (HR 0.64, 95% CI 0.08–5.26, *p* = 0.68) or PFS (HR 0.002, 95% CI 0.00–1.41, *p* = 0.06). Notably, these findings reflect substantial imprecision due to small event numbers.

## 4. Discussion

In this large single-institution cohort, the oncological outcomes of minimally invasive laparoscopic surgery were comparable to those of open radical surgery for early-stage cervical cancer. After adjustment for baseline imbalances using propensity-score matching, five-year OS and PFS were nearly identical between the LAP and the open groups (95.8% vs. 95.5% for OS and 92.3% vs. 93.8% for PFS), with no statistically significant differences observed. Subgroup analyses stratified by tumor size further demonstrated that LAP did not compromise survival in either small (≤2 cm) or larger (>2 cm) tumors, with similarly favorable five-year OS and PFS in both groups. Consistent with these findings, multivariable Cox regression analysis revealed that surgical approach was not an independent prognostic factor for OS or PFS.

Since the initial report by Nezhat in 1992 describing laparoscopic RH with pelvic and para-aortic lymphadenectomy [17], this approach has gained widespread use in gynecologic oncology as a minimally invasive alternative to open surgery for early-stage cervical cancer. Early observational studies consistently showed perioperative benefits of minimally invasive RH, such as less blood loss, shorter hospital stays, and faster recovery, without compromising cancer outcomes [4,18,19,20,21,22,23,24]. However, the pivotal LACC randomized trial raised substantial concerns regarding the oncologic safety of MIS RH. In this study of 631 women with stage IA1 (with LVSI), IA2, and IB1 cervical cancer, the 4.5-year disease-free survival was notably lower in the minimally invasive group compared with the open surgery arm (86.0% vs. 96.5%; difference −10.6 percentage points; 95% CI −16.4 to −4.7). Minimally invasive surgery was also linked to an increased risk of recurrence or death (HR 3.74, 95% CI 1.63–8.58) and higher all-cause mortality (HR 6.00, 95% CI 1.77–20.30) [5]. Supporting these findings, a large U.S. population-based analysis likewise showed reduced survival among patients undergoing MIS RH, with four-year mortality of 9.1% versus 5.3% for open surgery (HR 1.65) in women with stage IA2–IB1 disease. An interrupted time-series analysis revealed that, after adopting MIS techniques post-2006, four-year relative survival declined by 0.8% annually [25]. These findings raised concerns about the oncologic safety of minimally invasive radical hysterectomy and prompted further trials and studies worldwide. Recently, updated data from the LACC trial showed significantly poorer survival with MIS, with 4.5-year OS at 90.6% versus 96.2% for open RH (HR for death of any cause 2.71 [95% CI, 1.32 to 5.59]) [26]. As a result, many institutions and international guidelines now recommend open RH as the preferred surgical approach for early-stage cervical cancer [27,28].

The inferior survival outcomes associated with MIS RH, as reported in the LACC trial and subsequent population-based studies, have prompted extensive debate and investigation. Several hypotheses have been proposed to explain these findings, focusing primarily on technical, procedural, and surgeon-related factors. The most commonly discussed mechanisms include the following: (1) inadequate surgical radicality due to the steep learning curve of laparoscopic radical hysterectomy, (2) tumor spillage or dissemination related to the use of uterine manipulators, (3) intraperitoneal tumor exposure and implantation resulting from intracorporeal colpotomy performed under carbon dioxide (CO_2_) pneumoperitoneum, and (4) improper case selection [29,30]. Each of these factors may compromise oncologic integrity through different mechanisms.

Laparoscopic radical hysterectomy is a technically demanding procedure that requires a long learning curve to achieve oncologic outcomes equivalent to open surgery [30,31]. Insufficient experience may lead to inadequate parametrial resection, incomplete vaginal cuff excision, and suboptimal lymphadenectomy, particularly in larger tumors (>2 cm) [32]. Furthermore, early in the learning curve, surgeons may prioritize minimally invasive technical performance over strict adherence to oncologic principles, potentially contributing to inferior survival outcomes. Previous studies indicate that surgeons usually need to perform at least 40–50 procedures to attain the surgical proficiency necessary for optimal radicality and positive oncological outcomes [33,34]. These findings highlight that surgical experience, mentorship, and procedural standardization are crucial determinants of oncologic safety in MIS RH. At our institution, LAP RH was performed exclusively by surgeons with adequate experience, each having completed more than 200 radical procedures. The importance of the learning curve in shaping surgical outcomes was also evident. When comparing the first five years of performing LAP RH (2003–2008) with the subsequent decade (2009–2019), we observed substantial improvements in perioperative outcomes. Operative time decreased significantly (median 467 vs. 320 min, *p* = 0.001), estimated blood loss was markedly reduced (450 vs. 150 mL, *p* < 0.001), and complication rates dropped from 27.6% to 3.8% (Table S7). These findings highlight the progressive refinement of surgical techniques and the increasing proficiency of surgeons over time.

The uterine manipulator, often used in laparoscopic procedures to enhance visualization and facilitate exposure, has been implicated theoretically as a potential source of tumor cell dissemination by disrupting the cervical tumor, creating broken tumor fragments, and promoting exfoliation of malignant cells into the peritoneal cavity [35,36]. Another plausible explanation for the inferior survival outcomes associated with MIS RH involves the conduct of colpotomy within the pneumoperitoneum environment. Performing intracorporeal colpotomy under CO_2_ insufflation exposes the cervical tumor directly to circulating gas, potentially causing aerosolization and implantation of exfoliated cancer cells onto the peritoneal surfaces, especially when using the Trendelenburg position typically employed during laparoscopic procedures. Experimental models have shown that CO_2_ pneumoperitoneum can facilitate peritoneal tumor implantation, and clinical studies have identified intracorporeal colpotomy as an independent risk factor for recurrence following MIS RH. The resulting recurrence pattern differs from that of open surgery, with pelvic or peritoneal recurrences predominating instead of local vault failures [37,38]. Although definitive evidence remains limited, many experts now recommend avoiding intrauterine manipulators in cervical cancer surgery, using alternative uterine traction/manipulation techniques, performing vaginal colpotomy outside the pneumoperitoneum, sealing the vaginal cuff before opening, minimizing CO_2_ exposure, and performing thorough pelvic irrigation before closing. Kanao et al. demonstrated that meticulous avoidance of tumor manipulation during laparoscopic radical hysterectomy using the “no-look no-touch” technique can preserve oncologic safety. In a retrospective cohort comparing 80 women who underwent total laparoscopic radical hysterectomy using the no-look, no-touch technique with 83 women treated via open abdominal radical hysterectomy for stage IB1 cervical cancer (<4 cm), oncologic outcomes were similar between groups. The laparoscopic approach demonstrated comparable disease-free and overall survival while offering benefits such as shorter operative time, reduced blood loss, and shorter hospitalization. This technique integrates four measures designed to limit tumor exposure: preoperative creation of a vaginal cuff, avoidance of uterine manipulators, minimal manipulation of the cervix, and retrieval of the specimen in a containment bag to reduce the risk of intraperitoneal tumor spillage. Loco-regional recurrence rates were comparable between laparoscopic and open procedures [39]. Similarly, a large multicenter retrospective series by Köhler et al. examined 389 patients who underwent a combined laparoscopic–vaginal radical hysterectomy with transvaginal cuff closure and no uterine manipulator. With a median follow-up of 99 months, long-term outcomes remained excellent, with 4.5-year disease-free and overall survival rates of 95.8% and 97.8%, respectively. Recurrence was uncommon, and half were loco-regional. These findings suggest that when tumor spillage is prevented through a transvaginal approach and strict avoidance of uterine manipulation, MIS RH can achieve survival outcomes comparable to open surgery [40]. At our institution, LAP RH procedures were carried out with varying techniques. Forty-three patients (52.4%) had surgery without a uterine manipulator and with vaginal cuff closure (performed by CP), while the other half did not receive these preventive measures (performed by KC). Our analysis showed that employing these preventive (or no contamination) techniques was not significantly associated with improved OS and PFS. However, the small sample size of this subgroup analysis, the limited number of oncologic events, and correspondingly wide confidence intervals may preclude firm conclusions about the effect of these techniques. A recent systematic review and meta-analysis of six observational studies involving 2150 women found that even when the uterine manipulator was omitted, MIS RH remained associated with a higher risk of cancer recurrence compared with the open approach (HR 1.55, 95% CI 1.15–2.10) [41]. These findings suggest that while avoidance of uterine manipulation and vaginal cuff closure may reduce the risk of intraoperative tumor dissemination, other factors, such as surgical radicality or case selection, may still contribute to the oncological outcomes observed with MIS RH.

Notably, half of the patients in this cohort had undergone prior conization, which could have attenuated the impact of the tumor-protective measures. Given this observation, existing evidence regarding the role of preoperative conization warrants consideration. Previous studies have suggested that preoperative conization may contribute to oncologic benefits in early-stage cervical cancer. Bizzarri et al. demonstrated that preoperative conization was independently associated with a lower recurrence risk (five-year disease-free survival 89.8% vs. 80.0%, *p* = 0.01) and reduced need for adjuvant treatment in patients with early-stage cervical cancer who underwent RH, potentially through cytoreduction of tumor at the cervix, thereby minimizing intraperitoneal tumor spillage during minimally invasive surgery [42]. In addition, a recent propensity-score matched study from our institution assessing the association between conization prior to RH and survival outcomes in early-stage cervical cancer showed significantly improved overall survival among patients who underwent conization (log-rank *p* = 0.03), supporting the concept that conization may provide both biological and technique-related protection [43]. These findings have raised interest in identifying potential indications, procedural characteristics (e.g., method, cone depth, and margin status), and patient subgroups most likely to derive benefits, as well as evaluating whether conization could serve as a risk-modifying strategy in minimally invasive radical hysterectomy.

Following the LACC trial, many investigators have attempted to define appropriate selection criteria for MIS RH, with tumor size among the most examined factors. A retrospective study from West China Second University Hospital, including 678 women with early-stage cervical cancer, compared outcomes between laparoscopic (n = 255) and open (n = 423) radical hysterectomy. Overall and progression-free survival did not differ significantly between the two approaches in the entire cohort. However, subgroup analysis revealed that laparoscopic surgery was associated with significantly poorer overall survival in patients with tumors > 4 cm, whereas those with tumors ≤ 4 cm achieved better survival outcomes than the open group. Multivariate Cox regression confirmed that FIGO stage, histologic type, parametrial invasion, and pelvic lymph node metastasis, rather than surgical approach, were independent prognostic factors for both OS and PFS. These results suggest that minimally invasive radical hysterectomy may be an appropriate option for selected patients with smaller tumors (≤4 cm) but should be avoided in those with larger disease burden [44]. A recent multi-institutional retrospective study involving 815 patients across academic centers found that MIS RH was associated with a significantly higher risk of recurrence compared with open surgery, even after adjusting for confounding factors (adjusted HR 1.88, 95% CI 1.04–3.25). Notably, this increased recurrence risk persisted among patients with tumors ≤ 2 cm, with recurrence rates of 8.8% versus 2.4% for MIS and open approaches, respectively, and a hazard ratio of 2.83 (95% CI 1.10–7.18) in the propensity-matched analysis. Tumor size, grade, and adjuvant radiation were additional independent predictors of recurrence, whereas prior conization was associated with a significantly reduced risk (aHR 0.40, 95% CI 0.23–0.71) [45]. Our findings also support the importance of tumor size as an independent prognostic factor, regardless of surgical approach. In our multivariable analysis, pathological tumor size was one of the strongest independent prognostic factors, associated with OS and PFS. Nevertheless, in our subgroup analysis stratified at the 2 cm cut-off, survival outcomes were comparable between LAP and open RH. These findings suggest that, in our cohort, the survival disadvantage associated with LAP was not evident even among patients with larger tumors.

The strengths of our study include the use of a large, single-center cohort with uniform surgical, pathological, and survival assessment, minimizing variability in practice patterns. To minimize confounding, we applied rigorous propensity-score matching that incorporated a wide spectrum of clinically relevant variables, encompassing patient demographics, tumor characteristics, disease extent, and treatment-related factors. This robust adjustment allowed a well-balanced comparison between groups and strengthened the validity of our outcome analyses. Moreover, our study incorporated the evaluation of surgical technique, enabling exploration of its potential impact on survival outcomes within the minimally invasive cohort.

Our study also has certain limitations. First, its retrospective design can introduce bias. Second, several potentially relevant variables, such as tumor grade, LVSI, and depth of stromal invasion, were excluded from the propensity-score model due to missing data rates exceeding 20%, which may also introduce bias. Third, the significantly smaller sample size of the LAP group compared with the open group may have limited the statistical power to detect small but important differences in survival outcomes. Finally, as this was a single-institution analysis, the findings may not be fully generalizable to other settings with different patient populations, surgical expertise, or perioperative practices.

Despite these limitations, our study provides clinically meaningful insights. The findings from our institution can be applied to patient counseling when discussing the choice of surgical approach. In selected cases, MIS may be considered a safe alternative to open radical hysterectomy, as our data did not demonstrate inferior survival outcomes with the LAP. Moreover, if larger sample sizes become available in future analyses, it may be possible to identify additional prognostic factors associated with worse outcomes and refine patient selection criteria. Thus, our results support the role of MIS as a viable surgical option in carefully selected patients, while underscoring the importance of ongoing evaluation in our local context.

Future research should focus on prospective, multi-center studies or randomized controlled trials to confirm the effect of surgical approach on OS and PFS. The studies will need to carefully control for surgical technique and surgeon expertise, as these factors appear to influence outcomes in minimally invasive radical hysterectomy.

## 5. Conclusions

This single-institution propensity score–matched analysis shows that, when performed by experienced surgeons following standardized protocols, laparoscopic radical hysterectomy with pelvic lymphadenectomy can achieve long-term oncological outcomes comparable to those of open surgery. This result supports the cautious use of a laparoscopic approach in appropriately selected patients, particularly those with small-volume tumors and in settings where surgeons can ensure adequate radicality of the procedure through standardized techniques and possess substantial operative expertise. Shared decision-making remains essential, and patients should be thoroughly counseled regarding the potential risks and benefits of each surgical approach.

## Figures and Tables

**Table 1:** Baseline characteristics of patients before and after propensity-score matching.

Variable	Before Matching			After Matching		
	LAP (n = 82)	Open (n = 1162)	*p*-Value	LAP (n = 72)	Open (n = 279)	*p*-Value
Age: median (IQR), years	46 (41–54)	47 (41–53)	0.49	46 (41–53)	46 (40–51)	0.33
Parity: median (IQR)	2 (1–2)	2 (1–2)	0.91	2 (1–2)	2 (1–2)	0.70
HIV	0 (0.0%)	9 (0.8%)	1.00	0 (0.0%)	3 (1.1%)	1.00
Clinical size: median (IQR), cm	1.0 (0.0–2.5)	2.0 (0.0–3.0)	<0.001 *	1.0 (0.0–2.5)	1.5 (0.0–3.0)	0.20
Clinical size (Group)			0.07			0.78
≤2 cm	45 (68.2%)	479 (55.7%)		45 (70.3%)	149 (67.4%)	
>2 cm	21 (31.8%)	381 (44.3%)		19 (29.7%)	72 (32.6%)	
Pathology size: median (IQR), cm	1.2 (0.5–2.5)	2.0 (0.9–3.5)	<0.001 *	1.3 (0.7–2.5)	1.1 (0.7–2.3)	0.77
Pathology size (Group)			0.32			0.15
≤2 cm	39 (59.1%)	445 (51.9%)		39 (60.9%)	158 (71.5%)	
>2 cm	27 (40.9%)	413 (48.1%)		25 (39.1%)	63 (28.5%)	
Prior conization	42 (51.2%)	448 (38.6%)	0.03 *	36 (50.0%)	145 (52.0%)	0.87
Radical hysterectomy			0.49			0.77
Type B	5 (6.2%)	107 (9.2%)		4 (5.6%)	14 (5.0%)	
Type C	75 (93.8%)	1052 (90.8%)		68 (94.4%)	265 (95.0%)	
Histological type			0.65			1.00
Squamous cell carcinoma	57 (69.5%)	790 (68.0%)		52 (72.2%)	202 (72.4%)	
Adenocarcinoma	21 (25.6%)	283 (24.4%)		17 (23.6%)	65 (23.3%)	
Adenosquamous carcinoma	4 (4.9%)	89 (4.9%)		3 (4.2%)	12 (4.3%)	
Histological grade			0.63			0.07
Well differentiated	17 (27.9%)	235 (25.5%)		17 (29.3%)	51 (25.1%)	
Moderately differentiated	32 (52.5%)	537 (58.4%)		30 (51.7%)	133 (65.5%)	
Poorly differentiated	12 (19.7%)	148 (16.1%)		11 (19.0%)	19 (9.4%)	
Depth of stromal invasion			0.10			0.52
Inner1/3	15 (25.4%)	201 (20.9%)		14 (25.5%)	67 (31.8%)	
Middle1/3	16 (27.1%)	174 (18.1%)		16 (29.1%)	48 (22.7%)	
Outer1/3	28 (47.5%)	586 (61.0%)		25 (45.5%)	96 (45.5%)	
Presence of LVSI	29 (43.9%)	597 (58.1%)	0.03 *	25 (41.0%)	119 (50.2%)	0.25
Vaginal metastasis			0.89			0.70
Positive HSIL	5 (6.2%)	85 (7.3%)		5 (6.9%)	19 (6.8%)	
Positive CA	10 (12.3%)	154 (13.3%)		9 (12.5%)	26 (9.3%)	
Vaginal margin			0.87			0.72
Positive HSIL	5 (6.2%)	66 (5.7%)		5 (6.9%)	16 (5.7%)	
Positive CA	2 (2.5%)	47 (4.1%)		2 (2.8%)	5 (1.8%)	
Parametrial metastasis	11 (13.6%)	234 (20.2%)	0.19	11 (15.3%)	48 (17.2%)	0.83
Parametrial margin	0 (0.0%)	14 (1.2%)	1.00	0 (0.0%)	0 (0.0%)	1.00
Pelvic lymph node metastasis	11 (13.4%)	205 (17.6%)	0.41	9 (12.5%)	40 (14.3%)	0.83
Adnexal metastasis			0.01 *			0.58
Positive	0 (0.0%)	13 (1.1%)		0 (0.0%)	0 (0.0%)	
Not examined	28 (34.1%)	224 (19.3%)		26 (36.1%)	91 (32.6%)	
Uterine corpus metastasis			0.15			0.80
Positive HSIL	4 (5.1%)	118 (10.2%)		4 (5.6%)	20 (7.2%)	
Positive CA	0 (0.0%)	27 (2.3%)		0 (0.0%)	0 (0.0%)	
Surgical stage (FIGO 2018)			0.29			0.95
IA1 with positive LVSI or positive cone margin	8 (9.8%)	70 (6.0%)		4 (5.6%)	21 7.5%)	
IA2	5 (6.1%)	62 (5.3%)		4 (5.6%)	20 (7.2%)	
IB1	36 (43.9%)	375 (32.3%)		35 (48.6%)	133 (47.7%)	
IB2	11 (13.4%)	211 (18.2%)		10 (13.9%)	28 (10.0%)	
IB3	1 (1.2%)	52 (4.5%)		1 (1.4%)	3 (1.1%)	
IIA1	3 (3.7%)	62 (5.3%)		3 (4.2%)	7 (2.5%)	
IIA2	1 (1.2%)	10 (0.9%)		0 (0.0%)	1 (0.4%)	
IIB	6 (7.3%)	115 (9.9%)		6 (8.3%)	26 (9.3%)	
IIIC1	11 (13.4%)	205 (17.6%)		9 (12.5%)	40 (14.3%)	
Postoperative chemotherapy	16 (19.8%)	382 (32.9%)	0.01 *	16 (22.2%)	75 (26.9%)	0.51
Postoperative radiation	21 (27.6%)	451 (41.4%)	0.02 *	20 (27.8%)	89 (31.9%)	0.60

* Statistically significant *p* < 0.05. LAP: Laparoscopic, IQR: interquartile range, HIV: human immunodeficiency virus, LVSI: lymphovascular space invasion, HSIL: high-grade squamous intraepithelial lesion, CA: carcinoma, FIGO: International Federation of Gynecology and Obstetrics.

**Table 2:** Comparison of oncological outcomes between the LAP and the open RH groups.

Variable	Before Matching			After Matching		
	LAP (n = 82)	Open (n = 1162)	*p*-Value	LAP (n = 72)	Open (n = 279)	*p*-Value
Follow time: median (IQR), months	83.9 (50.3–115.8)	68.3 (32.7–119.2)	0.13	86.0 (50.9–122.5)	78.2 (37.7–140.9)	0.74
Recurrence	8 (9.8%)	107 (9.2%)	1.00	5 (6.9%)	17 (6.1%)	0.79
Site of recurrence			0.09			0.26
No	74 (90.2%)	1053 (90.6%)		67 (93.1%)	262 (93.9%)	
Pelvis	4 (4.9%)	49 (4.2%)		3 (4.2%)	7 (2.5%)	
Distant metastasis	0 (0.0%)	38 (3.3%)		0 (0.0%)	7 (2.5%)	
Pelvis + Distant metastasis	4 (4.9%)	22 (1.9%)		2 (2.8%)	3 (1.1%)	
Death	12 (14.6%)	170 (14.6%)	1.00	8 (11.1%)	31 (11.1%)	1.00

LAP, Laparoscopic, IQR, interquartile range.

**Table 3:** Multivariable Cox regression analysis for overall and progression-free survival in the entire study cohort.

Variable	Overall Survival			Progression-Free Survival		
	Hazard Ratio	95% Confidence Interval	*p*-Value	Hazard Ratio	95% Confidence Interval	*p*-Value
Age (years)	1.03	1.00–1.05	0.02 *	1.01	0.99–1.04	0.30
Parity	1.03	0.91–1.17	0.62	0.77	0.62–0.95	0.02 *
HIV						
Negative	1.00	Ref		1.00	Ref	
Positive	3.72	0.49–28.06	0.20	1.67	0.22–13.01	0.62
Prior conization						
No	1.00	Ref		1.00	Ref	
Yes	0.76	0.49–1.16	0.20	0.89	0.47–1.66	0.71
Route of surgery						
Open	1.00	Ref		1.00	Ref	
LAP	0.83	0.40–1.71	0.61	1.12	0.44–2.84	0.82
Radical hysterectomy						
Type B	1.00	Ref		1.00	Ref	
Type C	0.66	0.33–1.32	0.24	0.63	0.26–1.54	0.31
Histological type						
Squamous cell carcinoma	1.00	Ref		1.00	Ref	
Adenocarcinoma	0.99	0.66–1.48	0.96	1.52	0.95–2.45	0.08
Adenosquamous carcinoma	1.77	1.09–2.88	0.02 *	2.08	1.14–3.82	0.02 *
Pathological tumor size	1.34	1.16–1.55	<0.001 *	1.69	1.42–2.03	<0.001 *
Parametrial metastasis						
Negative	1.00	Ref		1.00	Ref	
Positive	1.01	0.65–1.57	0.96	1.20	0.66–2.18	0.55
Parametrial margin						
Negative	1.00	Ref		1.00	Ref	
Positive	0.64	0.15–2.75	0.54	0.44	0.06–3.42	0.43
Pelvic lymph node metastasis						
Negative	1.00	Ref		1.00	Ref	
Positive	1.60	1.02–2.51	0.04 *	1.23	0.68–	0.50
Adnexal metastasis						
Negative	1.00	Ref		1.00	Ref	
Positive	1.11	0.37–3.30	0.85	0.86	0.27–2.71	0.79
Not examined	0.81	0.50–1.34	0.42	1.28	0.72–2.27	0.40
Uterine corpus metastasis						
Negative	1.00	Ref		1.00	Ref	
Positive HSIL	0.85	0.52–1.39	0.52	1.35	0.76–2.40	0.30
Positive CA	0.83	0.25–2.71	0.76	2.27	0.67–7.65	0.19
Vaginal metastasis						
Negative	1.00	Ref		1.00	Ref	
Positive HSIL	0.69	0.33–1.44	0.32	0.64	0.25–1.68	0.37
Positive CA	1.53	1.01–2.31	0.05 *	1.55	0.89–2.72	0.12
Vaginal margin						
Negative	1.00	Ref		1.00	Ref	
Positive	1.21	0.62–2.36	0.58	1.95	0.88–4.31	0.10
Postoperative chemotherapy						
No	1.00	Ref		1.00	Ref	
Yes	1.12	0.67–1.89	0.66	1.01	0.50–2.05	0.97
Postoperative radiation						
No	1.00	Ref		1.00	Ref	
Yes	1.01	0.61–1.69	0.97	0.61	0.31–1.22	0.16
Number of resected pelvic lymph nodes	1.00	098–1.02	0.67	0.99	0.97–1.01	0.32

* Statistically significant *p* < 0.05. HIV: human immunodeficiency virus, HSIL: high-grade squamous intraepithelial lesion, CA: carcinoma.

## Data Availability

The data presented in this study are available on request from the corresponding author (The data are not publicly available due to privacy or ethical restrictions).

## Supplementary Materials

The following supporting information can be downloaded at https://www.mdpi.com/article/10.3390/cancers17243960/s1, Figure S1: Kaplan–Meier curve comparing overall survival and progression-free survival between the LAP and the open RH groups (before matching); Table S1: Summary of missing data; Table S2: Baseline characteristics of patients before and after propensity-score matching (Tumors ≤ 2 cm); Table S3: Comparison of oncological outcomes between the LAP and the open RH groups (Tumors ≤ 2 cm); Table S4: Baseline characteristics of patients before and after propensity-score matching (Tumors > 2 cm); Table S5: Comparison of oncological outcomes between the LAP and the open RH groups (Tumors > 2 cm); Table S6: Multivariable Cox regression analysis for overall survival and progression-free survival in the laparoscopic radical hysterectomy group; Table S7: Perioperative outcomes of laparoscopic radical hysterectomy in early vs. later period.
