# Supplementary material for: Long-Term Oncological Outcomes of Laparoscopic Versus Open Radical Surgery in Early-Stage Cervical Cancer: A Propensity Score–Matched Analysis
Source: Cancers (Basel). 2025 Dec 11;17(24):3960. doi: 10.3390/cancers17243960 (PMC12731032; doi:10.3390/cancers17243960)
Supplement: Supplementary file 1 [file cancers-17-03960-s001.zip › Table S1.pdf]

Table S1. Summary of missing data.

Variable	Proportion of missingness (%)
Age	0.00
Parity	0.00
HIV status	0.16
Clinical size	1.27
Pathology size	0.56
Prior conization	0.00
Radical hysterectomy class	0.48
Histological type	0.00
Histological grade	21.51
Depth of stromal invasion	18.49
Presence of LVSI	12.54
Vaginal metastasis	0.48
Vaginal margin	0.40
Parametrial metastasis	0.56
Parametrial margin	0.32
Pelvic lymph node metastasis	0.00
Adnexal metastasis	0.32
Uterine corpus metastasis	0.40
Postoperative chemotherapy	0.24
Postoperative radiation	0.16
