# Supplementary material for: Long-Term Oncological Outcomes of Laparoscopic Versus Open Radical Surgery in Early-Stage Cervical Cancer: A Propensity Score–Matched Analysis
Source: Cancers (Basel). 2025 Dec 11;17(24):3960. doi: 10.3390/cancers17243960 (PMC12731032; doi:10.3390/cancers17243960)
Supplement: Supplementary file 1 [file cancers-17-03960-s001.zip › Table S2.pdf]

Table S2. Baseline characteristics of patients before and after propensity-score matching (Tumors ≤ 2 cm).

Variable	Before matching			After matching		
	LAP (n =53)	Open (n =589)	*p*-value	LAP (n =47)	Open (n =188)	*p*-value
**Age:** mean (SD), years	47.2 (8)	47.9 (8.4)	0.575	48 (8)	45.7 (7.7)	0.081
**Parity:** median (IQR)	2 (1-2)	2 (1-2)	0.395	2 (1-2)	2 (1-2)	0.575
**HIV**	0 (0.0%)	4 (0.7%)	1.000	0 (0.0%)	2 (1.1%)	1.000
**Clinical size:** median (IQR), cm	0 (0-1)	0 (0-1.5)	0.040*	0 (0-1)	0 (0-1.5)	0.456
**Pathology size:** median (IQR), cm	0.8 (0-1.2)	0.9 (0.3-1.4)	0.078	0.8 (0.4-1.3)	0.8 (0-1.2)	0.724
**Prior conization**	38 (71.7%)	394 (66.9%)	0.575	32 (68.1%)	139 (73.9%)	0.533
**Radical hysterectomy**			0.173			0.200
Type B	4 (7.7%)	93 (15.8%)		3 (6.4%)	5 (2.7%)	
Type C	48 (92.3%)	495 (84.2%)		44 (93.6%)	183 (97.3%)	
**Histological type**			0.683			0.362
Squamous cell carcinoma	34 (64.2%)	396 (67.2%)		31 (66.0%)	141 (75.0%)	
Adenocarcinoma	17 (32.1%)	158 (26.8%)		15 (31.9%)	43 (22.9%)	
Adenosquamous carcinoma	2 (3.8%)	35 (5.9%)		1 (2.1%)	4 (2.1%)	
**Histological grade**			0.131			0.012*
Well differentiated	14 (40.0%)	117 (30.8%)		14 (40.0%)	31 (28.7%)	
Moderately differentiated	14 (40.0%)	215 (56.6%)		14 (40.0%)	70 (64.8%)	
Poorly differentiated	7 (20.0%)	48 (12.6%)		7 (20.0%)	7 (6.5%)	
**Depth of stromal invasion**			0.082			0.341
Inner1/3	15 (48.4%)	176 (44.6%)		14 (46.7%)	59 (51.8%)	
Middle1/3	12 (38.7%)	100 (25.3%)		12 (40.05)	31 (27.2%)	
Outer1/3	4 (12.9%)	119 (30.1%)		4 (13.3%)	24 (21.1%)	
**Presence of LVSI**	9 (23.7%)	181 (39.1%)	0.088	8 (22.2%)	44 (31.9%)	0.356
**Vaginal metastasis**			0.517			1.000
Positive HSIL	1 (1.9%)	31 (5.3%)		1 (2.1%)	5 (2.7%)	
Positive CA	1 (1.9%)	28 (4.8%)		1 (2.1%)	5 (2.7%)	
**Vaginal margin**			0.635			1.000
Positive HSIL	1 (1.9%)	25 (4.3%)		1 (2.1%)	6 (3.2%)	
Positive CA	0 (0.0%)	14 (2.4%)		0 (0.0%)	0 (0.0%)	
**Parametrial metastasis**	2 (3.8%)	35 (6.0%)	0.760	2 (4.3%)	7 (3.7%)	1.000
**Parametrial margin**	0 (0.0%)	0 (0.0%)	1.000	0 (0.0%)	0 (0.0%)	1.000
**Pelvic lymph node metastasis**	2 (3.8%)	41 (7.0%)	0.567	2 (4.3%)	7 (3.7%)	1.000
**Adnexal metastasis**			0.155			0.595
Positive	0 (0.0%)	3 (0.5%)		0 (0.0%)	0 (0.0%)	
Not examined	16 (30.2%)	114 (19.4%)		15 (31.9%)	53 (28.2%)	

Variable	Before matching			After matching		
	**LAP (n =53)**	**Open (n =589)**	***p*-value**	**LAP (n =47)**	**Open (n =188)**	***p*-value**
**Uterine corpus metastasis**			0.301			1.000
Positive HSIL	0 (0.0%)	16 (2.7%)		0 (0.0%)	0 (0.0%)	
Positive CA	0 (0.0%0	19 (3.2%)		0 (0.0%)	0 (0.0%)	
**Postoperative chemotherapy**	4 (7.5%)	88 (14.9%)	0.205	4 (8.5%)	10 (5.3%)	0.488
**Postoperative radiation**	4 (8.3%)	116 (20.3%)	0.068	4 (8.5%)	19 (10.1%)	1.000
