# Supplementary material for: Long-Term Oncological Outcomes of Laparoscopic Versus Open Radical Surgery in Early-Stage Cervical Cancer: A Propensity Score–Matched Analysis
Source: Cancers (Basel). 2025 Dec 11;17(24):3960. doi: 10.3390/cancers17243960 (PMC12731032; doi:10.3390/cancers17243960)
Supplement: Supplementary file 1 [file cancers-17-03960-s001.zip › Table S3.pdf]

**Table S3.** Comparison of oncological outcomes between the LAP and the open RH groups (Tumors ≤ 2 cm).

| Variable | Before matching | | | After matching | | |
|---|---|---|---|---|---|---|
| | **LAP (n =53)** | **Open (n =589)** | ***p*-value** | **LAP (n =47)** | **Open (n =188)** | ***p*-value** |
| **Follow time:** median (IQR), months | 94.8 (60-125.8) | 73.8 (36.3-126.9) | 0.066 | 94.8 (57.4-133.8) | 80.8 (39.8-141) | 0.365 |
| **Recurrence** | 1 (1.9%) | 18 (3.1%) | 1.000 | 0 (0.0%) | 4 (2.1%) | 0.586 |
| **Site of recurrence** | | | 1.000 | | | 0.669 |
| No | 52 (98.1%) | 570 (96.8%) | | 47 (100.0%) | 183 (97.3%) | |
| Pelvis | 1 (1.9%) | 15 (2.5%) | | 0 (0.0%) | 4 (2.1%) | |
| Distant metastasis | 0 (0.0%) | 4 (0.7%) | | 0 (0.0%) | 1 (0.5%) | |
| Pelvis + Distant metastasis | 0 (0.0%) | 0 (0.0%) | | 0 (0.0%) | 0 (0.0%) | |
| **Death** | 3 (5.7%) | 49 (8.3%) | 0.791 | 3 (6.4%) | 12 (6.4%) | 1.000 |
