# Supplementary material for: Long-Term Oncological Outcomes of Laparoscopic Versus Open Radical Surgery in Early-Stage Cervical Cancer: A Propensity Score–Matched Analysis
Source: Cancers (Basel). 2025 Dec 11;17(24):3960. doi: 10.3390/cancers17243960 (PMC12731032; doi:10.3390/cancers17243960)
Supplement: Supplementary file 1 [file cancers-17-03960-s001.zip › Table S4.pdf]

Table S4. Baseline characteristics of patients before and after propensity-score matching (Tumors > 2 cm).

Variable	Before matching			After matching		
	LAP (n =28)	Open (n =568)	*p*-value	LAP (n =24)	Open (n =90)	*p*-value
**Age:** median (IQR), years	45 (38-51.5)	47 (40-53)	0.382	45 (38-51.5)	44 (38.2-52.8)	0.989
**Parity:** median (IQR)	2 (1-3)	2 (1-2)	0.531	2 (1.8-2.2)	2 (1-2)	0.746
**HIV**	0 (0.0%)	5 (0.9%)	1.000	0 (0.0%)	1 (1.1%)	1.000
**Clinical size:** median (IQR), cm	3 (2-3)	3 (3-4)	0.003*	3 (2-3)	3 (2-3)	0.216
**Pathology size:** median (IQR), cm	3 (2.5-3.6)	3.5 (2.8-4.3)	0.006*	3 (1.5-3.6)	3 (1.5-3.7)	0.479
**Prior conization**	4 (14.3%)	50 (8.8%)	0.308	4 (16.7%)	13 (14.4%)	0.754
**Radical hysterectomy**			0.518			0.511
Type B	1 (3.6%)	14 (2.5%)		1 (4.2%)	2 (2.2%)	
Type C	27 (96.4%)	554 (97.5%)		23 (95.8%)	88 (97.8%)	
**Histological type**			0.704			1.000
Squamous cell carcinoma	22 (78.6%)	391 (68.6%)		20 (83.3%)	72 (80.0%)	
Adenocarcinoma	4 (14.3%)	124 (21.8%)		2 (8.3%)	10 (11.1%)	
Adenosquamous carcinoma	2 (7.1%)	53 (9.3%)		2 (8.3%)	8 (8.9%)	
**Histological grade**			0.499			0.773
Well differentiated	3 (11.5%)	117 (21.7%)		3 (13.6%)	9 (10.7%)	
Moderately differentiated	18 (69.2%)	321 (59.7%)		16 (72.7%)	65 (77.4%)	
Poorly differentiated	5 (19.2%)	100 (18.6%)		3 (13.6%)	10 (11.9%)	
**Depth of stromal invasion**			0.707			1.000
Inner1/3	0 (0.0%)	25 (4.4%)		0 (0.0%)	3 (3.4%)	
Middle1/3	4 (14.3%)	72 (12.8%)		4 (16.7%)	15 (16.9%)	
Outer1/3	24 (85.7%)	466 (82.8%)		20 (83.3%)	71 (79.8%)	
**Presence of LVSI**	20 (71.4%)	416 (74.0%)	0.933	17 (70.8%)	71 (79.8%)	0.510
**Vaginal metastasis**			0.215			0.953
Positive HSIL	4 (14.3%)	54 (9.5%)		4 (16.7%)	16 (17.8%)	
Positive CA	9 (32.1%)	125 (22.0%)		7 (29.2%)	29 (32.2%)	
**Vaginal margin**			0.212			1.000
Positive HSIL	4 (14.3%)	41 (7.2%)		4 (16.7%)	15 (16.7%)	
Positive CA	2 (7.1%)	32 (5.6%)		2 (8.3%)	7 (7.8%)	
**Parametrial metastasis**	9 (32.1%)	198 (35.0%)	0.917	9 (37.5%)	30 (33.3%)	0.889
**Parametrial margin**	0 (0.0%)	14 (2.5%)	1.000	0 (0.0%)	0 (0.0%)	1.000
**Pelvic lymph node metastasis**	8 (28.6%)	161 (28.3%)	1.000	7 (29.2%)	29 (32.2%)	0.969
**Adnexal metastasis**			0.017*			1.000
Positive	0 (0.0%)	10 (1.8%)		0 (0.0%)	0 (0.0%)	
Not examined	12 (42.9%)	108 (19.1%)		10 (41.7%)	37 (41.1%)	

Variable	Before matching			After matching		
	LAP (n =28)	Open (n =568)	*p*-value	LAP (n =24)	Open (n =90)	*p*-value
**Uterine corpus metastasis**			0.864			1.000
Positive HSIL	4 (14.8%)	101 (17.8%)		4 (16.7%)	14 (15.6%)	
Positive CA	0 (0.0%)	8 (1.4%)		0 (0.0%)	0 (0.0%)	
**Postoperative chemotherapy**	12 (44.4%)	289 (51.1%)	0.635	12 (50.0%)	43 (47.8%)	1.000
**Postoperative radiation**	16 (59.3%)	330 (64.5%)	0.732	16 (66.7%)	66 (73.3%)	0.696
