# Supplementary material for: Long-Term Oncological Outcomes of Laparoscopic Versus Open Radical Surgery in Early-Stage Cervical Cancer: A Propensity Score–Matched Analysis
Source: Cancers (Basel). 2025 Dec 11;17(24):3960. doi: 10.3390/cancers17243960 (PMC12731032; doi:10.3390/cancers17243960)
Supplement: Supplementary file 1 [file cancers-17-03960-s001.zip › Table S5.pdf]

Table S5. Comparison of oncological outcomes between the LAP and the open RH groups. (Tumors > 2 cm).

Variable	Before matching			After matching		
	LAP (n =28)	Open (n =568)	*p*-value	LAP (n =24)	Open (n =90)	*p*-value
**Follow time:** median (IQR), months	74.8 (24.7-110.1)	63.2 (30.4-109.2)	0.714	76.3 (31.8-110.7)	73.9 (27.8-117.7)	0.846
**Recurrence**	7 (25.0%)	89 (15.7%)	0.191	5 (20.8%)	13 (14.4%)	0.529
**Site of recurrence**			0.028*			0.389
No	21 (75.0%)	478 (84.2%)		19 (79.2%)	77 (85.6%)	
Pelvis	3 (10.7%)	34 (6.0%)		2 (12.5%)	5 (5.6%)	
Distant metastasis	0 (0.0%)	34 (6.0%)		0 (0.0%)	4 (4.4%)	
Pelvis + Distant metastasis	4 (14.3%)	22 (3.9%)		2 (8.3%)	4 (4.4%)	
**Death**	8 (28.6%)	121 (21.3%)	0.499	5 (20.8%)	24 (26.7%)	0.750
