# Supplementary material for: Long-Term Oncological Outcomes of Laparoscopic Versus Open Radical Surgery in Early-Stage Cervical Cancer: A Propensity Score–Matched Analysis
Source: Cancers (Basel). 2025 Dec 11;17(24):3960. doi: 10.3390/cancers17243960 (PMC12731032; doi:10.3390/cancers17243960)
Supplement: Supplementary file 1 [file cancers-17-03960-s001.zip › Table S6.pdf]

Table S6. Multivariable Cox regression analysis for overall survival and progression-free survival in the laparoscopic radical hysterectomy group.

Variable	Overall survival			Progression-free survival		
	Hazard ratio	95% Confidence interval	p-value	Hazard ratio	95% Confidence interval	p-value
**Age, years**	1.05	0.95 – 1.18	0.335	0.91	0.71 – 1.16	0.440
**Radical hysterectomy**						
Type B	1.00	Ref		1.00	Ref	
Type C	0.06	0.00 – 1.17	0.063	8.21e-05	2.21e-08 – 0.30	0.025*
**Histological type**						
Squamous cell carcinoma	1.00	Ref		1.00	Ref	
Adenocarcinoma	4.41	0.72 – 27.11	0.110	0.06	0.29 – 1.28e+06	0.101
Adenosquamous carcinoma	0.93	0.04 – 21.55	0.966	3.53e+03	0.74 – 1.68e+07	0.059
**Pathological tumor size**	2.68	1.35 – 5.31	0.005*	61.08	0.64 – 5.87e+03	0.078
**Parametrial metastasis**						
Negative	1.00	Ref		1.00	Ref	
Positive	0.28	0.02 – 4.21	0.356	63.77	0.08 – 5.28e+04	0.225
**Pelvic lymph node metastasis**						
Negative	1.00	Ref		1.00	Ref	
Positive	3.70	0.44 – 31.28	0.230	0.03	4.57e-06 – 161.30	0.416
**Uterine corpus metastasis**						
Negative	1.00	Ref		1.00	Ref	
Positive HSIL	1.86	0.17 – 20.63	0.614	14.58	0.28 – 759.70	0.184
**Vaginal margin status**						
Negative	1.00	Ref		1.00	Ref	
Positive	0.19	0.01 – 4.37	0.299	0.08	9.94e-05 – 61.33	0.453
**No-contamination technique**						
No	1.00	Ref		1.00	Ref	
Yes	0.90	0.13 – 6.27	0.917	5.44	0.04 – 67.85	0.492
**Conization**						
No	1.00	Ref		1.00	Ref	
Yes	2.15	0.29 – 16.02	0.456	1.99e+03	0.01 – 5.06e+08	0.232
**Surgery period**						
2003 - 2008	1.00	Ref		1.00	Ref	
2009 - 2019	0.64	0.08 – 5.26	0.677	2.18e-03	3.36e-06 – 1.41	0.064

* Statistically significant $p < 0.05$

IQR; interquartile range, HSIL; high-grade squamous intraepithelial lesion
