# Supplementary material for: Long-Term Oncological Outcomes of Laparoscopic Versus Open Radical Surgery in Early-Stage Cervical Cancer: A Propensity Score–Matched Analysis
Source: Cancers (Basel). 2025 Dec 11;17(24):3960. doi: 10.3390/cancers17243960 (PMC12731032; doi:10.3390/cancers17243960)
Supplement: Supplementary file 1 [file cancers-17-03960-s001.zip › Table S7.pdf]

Table S7. Perioperative outcomes of laparoscopic radical hysterectomy in early vs later period.

Variable	2003-2008 (n = 29)	2009-2019 (n = 53)	p-value
Operative time: median (IQR), minutes	467 (430-523)	320 (262-422)	0.001*
Estimated blood loss: median (IQR), ml	450 (400-745)	150 (100-320)	<0.001*
Complications			0.003*
No	21 (72.4%)	51 (96.2%)	
Yes	8 (27.6%)	2 (3.8%)	
Number of resected pelvic nodes: Mean (SD)	21 (8.9)	19 (7.2)	0.321

* Statistically significant p < 0.05

IQR; interquartile range, SD; standard deviation
